# Analyzing generic and branded substitution patterns in the Netherlands using prescription data

**DOI:** 10.1186/1472-6963-11-89

**Published:** 2011-04-27

**Authors:** Petros Pechlivanoglou, Willem Jan van der Veen, Jens H Bos, Maarten J Postma

**Affiliations:** 1 Unit of PharmacoEpidemiology and PharmacoEconomics (PE2), Department of Pharmacy, University of Groningen, A. Deusinglaan 1, 9713 AV Groningen, The Netherlands; 2 Department of General Practice, University Medical Centre Groningen, Hanzeplein 1 9713 GZ, Groningen, The Netherlands

## Abstract

**Background:**

As in other societies, pharmaceutical expenditures in the Netherlands are rising every year. As a consequence, needs for cost control are often expressed. One possible solution for cost control could come through increasing generic substitution by pharmacists. We aim to analyse the extent and nature of substitution in recent years and estimate the likelihood of generic or branded substitution in Dutch pharmacies in relation to various characteristics.

**Methods:**

We utilized a linked prescription dataset originating from a general practitioner (GP) and a pharmacy database, both from the northern Netherlands. We selected specific drugs of interest, containing about 55,000 prescriptions from 15 different classes. We used a crossed generalized linear mixed model to estimate the effects that certain patient and pharmacy characteristics as well as timing have on the likelihood that a prescription will eventually be substituted by the pharmacist.

**Results:**

Generic substitution occurred at 25% of the branded prescriptions. Generic substitution was more likely to occur earlier in time after patent expiry and to patients that were older and more experienced in their drug use. Individually owned pharmacies had a lower probability of generic substitution compared to chain pharmacies. Oppositely, branded substitution occurred in 10% of generic prescriptions and was positively related to the patients' experience in branded use. Individually owned pharmacies were more likely to substitute a generic drug to a branded compared to other pharmacies. Antidepressant and PPI prescriptions were less prone to generic and more prone to branded substitution.

**Conclusion:**

Analysis of prescription substitution by the pharmacist revealed strong relations between substitution and patient experience on drug use, pharmacy status and timing. These findings can be utilised to design further strategies to enhance generic substitution.

## Background

Despite the efforts of governments, insurance companies and health care providers to enhance cost control, health expenditures in the developed world rise every year. The main reasons for this trend are the ageing of the population, growing expectations regarding health by the society as well as the continuous improvement in health technologies [1]. Expenditures on pharmaceuticals are responsible for a major share in health expenditures and the percentage of pharmaceutical expenditures has been constantly rising for the last 30 years [1]. The Netherlands also faces the same trend of increasing health care and pharmaceutical costs. Pharmaceutical costs however, if expressed as a percentage of the total health expenditures are much lower in the Netherlands, compared to other major European economies such as Germany, UK and France [2]. Possibly, the relatively strict regulatory policy on the introduction and reimbursement of new drugs, the restrictive prescription policies (for example, regarding antibiotics) and the active stimulation of generic drug use might all be related to this phenomenon.

In particular, the potentials for cost savings due to generic drug use has been recognized relatively early in the Netherlands, where in 1988 already about a quarter of the prescriptions were prescribed using the generic name [3]. The Dutch government, in its effort to contain costs, would stimulate generic use through generic substitution; i.e. the delivery of a generic drug by the pharmacist when a branded drug is indicated on the GP prescription. Although generic substitution was already allowed, provided that the pharmacist would first consult the prescriber, in 1998 the Dutch law stimulated generic substitution further by relaxing this compulsory consultation. Nowadays, the Netherlands belongs to the group of countries with the highest proportion of generic use, with a market volume of generic drugs slightly above 50% of the total national drug volume [4].

The reasons for a pharmacist to embark on generic substitution vary, but might (partly) be related to financial considerations within the private pharmacist's own business and an awareness to enhance cost control in general. In particular, a pharmacist may dispense the generic instead of the branded drug because:

• at least until 2000, the pharmacist gained more profit through discounts offered from manufacturers of generic rather than of branded drugs [5];

• until 2003, a law aiming to stimulate generic use allowed the pharmacist to keep one third of the difference between the reference and actual price of the dispensed drug;

• there is no available stock of the branded product at the pharmacy;

• the pharmacist behaves cost consciously and is willing to adhere to governmental regulations and public preferences; and

• a branded prescription may not be (fully) reimbursed by the insurance company if the generic alternative is a lot cheaper, especially if there is a specific policy from the insurance company towards generics (for example, a preference policy regarding several drug classes has been endorsed by various insurance companies in the Netherlands).

Yet, despite the pressure of the government and the insurance companies on the broader use of generic drugs, the pharmaceutical expenditures in the Netherlands are still increasing [6]. One reason for this phenomenon is the relatively high level at which the producers still set the generic prices. Also, during the last years an increasing number of new branded drugs entered the pharmaceutical market, increasing the potentials for alternative means of treatment, branded prescribing and substitution between brands [7]. According to Gumbs et al [8], the Dutch society could benefit with a health expenditure reduction of 71 million Euros if branded statin prescribing would be generically substituted in current users who are eligible for receiving generic treatment. Finally, in daily pharmacy practice, branded substitution is sometimes observed, where a generic prescription is being filled with the branded drug. Reasons for this include the level of severity of the disease, the personal preferences of the patient, the financial profit of the pharmacist as well as existing or perceived adverse effects related to generics in specific disease types [9].

Although stimulation of generic substitution in the Netherlands was initiated already more than a decade ago, little has been published regarding its frequency in daily practice. The aim of this study was to analyse the extent of generic and branded substitution for a number of popular drug classes in the northern Netherlands and subsequently to quantify the effect that several patient and pharmacist characteristics, as well as the timing of prescriptions had on the likelihood of generic or branded substitution by the pharmacist.

## Methods

### Data Source

The dataset for the analyses was established after linking prescriptions from databases on GP prescribing and pharmacy delivering, for a set of different drug classes (each class containing one branded and one or more generic drugs of the same chemical entity). We selected drug classes from the four most prescribed drug groups in the Netherlands (statins, angiotensin-converting enzyme (ACE) inhibitors, proton pump inhibitors (PPIs) and antidepressants), where also at least one generic was available. Furthermore, we selected drug classes where the branded drug's patent expiry occurred within the period for which the linked databases could provide sufficient information, both prior and post expiry. Table 1 shows the drugs that were included, using these selection criteria.

**Table 1:** Drug groups included in the analysis, branded manufacturers between brackets and patent expiry dates

Drug name	Branded drug	Patent expiry date^a^
ACE Inhibitors		
Enalapril	Renitec^® ^[MSD]	13-12-1999
Lisinopril	Zestril^® ^[AstraZeneca]	8-10-2002
Ramipril	Tritace^® ^[Sanofi-Aventis]	13-1-2004
Quinapril	Acupril^® ^[Pfizer]	5-10-2004
Fosinopril	Newace^® ^[BMS]	4-7-2005
Antidepressants		
Fluoxetine	Prozac^® ^[Eli Lilly]	14-12-1999
Paroxetine	Seroxat^® ^[GSK]	12-7-2001
Citalopram	Cipramil^® ^[Lundbeck]	1-2-2002
Moclobemide	Aurorix^® ^[Roche]	4-4-2002
Mirtrazapine	Remeron^® ^[Shering-Plough]	13-9-2004
Sertraline	Zoloft^® ^[Pfizer]	28-10-2005
PPIs		
Omperazol	Losec^® ^[AstraZeneca]	18-4-2002
Lansoprazol	Prezal^® ^[Sanofi-Aventis]	19-12-2005
Statins		
Simvastatine	Zocor^® ^[MSD]	1-5-2003
Pravastatine	Selektine^® ^[BMS]	2-8-2004

^a ^Defined as the date of the first prescription encountered in IADB.nl

GP prescriptions were collected from the Registration Network Groningen (RNG) database [10,11], while the pharmacy dispensing information was collected from IADB.nl [12].

RNG has an annual average population size of about 30,000 patients. The RNG comprises three practices in the northern Netherlands and a total number of 17 participating GPs. All GPs routinely register the patient data electronically through a specific software package. Within the registered information, for example, date of entry to the practice, exit data (if applicable) and dates of visits are recorded, enabling analyses where person-based tracking in time is necessary, such as calculations of rates of presence and occurrence of episodes, disease and prescriptions (prevalence and incidence).

IADB.nl comprises the full populations of the main cities and some regional centers in the north of the Netherlands. The adherent population of IADB.nl is approximately 500,000 persons. The database was most recently updated to include prescription/dispensing data up to and including 01-01-2007.

### Linking Procedure

For the selected drugs, data on prescribing and dispensing were selected from RNG and IADB.nl from the time of patent expiry of each drug until the end of the last update of IADB.nl (01-01-2007). These data were subsequently linked to each other. The linking of the two datasets was based on the personal information of the patients (postal code, gender and date of birth). Thus, patients were matched in both databases on the basis of their date of birth, their gender and finally their postal code of residence. The linking procedure was performed under strict privacy rules and patient anonymity was ensured for the researchers involved. The combinations of these characteristics, together with the exact information on the Anatomic Therapeutic Chemical (ATC) code, enabled the identification of 96% of the RNG patients in IADB.nl.

### Variables Investigated

Through the linking procedure described above we obtained information on the drug type (branded or generic) that was initially prescribed and the type that was finally dispensed, linked with different patient and pharmacy characteristics as well as on information regarding the exact timing of the prescription.

The outcome studied was whether a prescription filled with the branded (generic) name by the GP was substituted with the generic (branded) during delivery from the pharmacist.

Past research has shown that the decision of generic or branded substitution can be influenced by drug price, drug class and patient, pharmacy and insurance company characteristics [7]. Here, we assumed that price should not be included as an explanatory characteristic in our analysis since copayment was not necessary for any of the studied drugs, and therefore the patient doesn't really notice the exact price level. Information regarding the additional profit that pharmacists might have gained through relevant discounts offered during the wholesaling process, although potentially relevant, was not included in the analysis as no data on this was available, neither in the linked dataset, nor outside of it. Further, since health insurance is compulsory in the Netherlands and all prescriptions were fully reimbursed by all health insurances, we did not investigate different substitution patterns among prescriptions potentially caused by different health insurance schemes. Therefore, for every prescription in the database we collected the following explanatory variables:

• A categorical variable indicating the drug group of the prescription (ACE inhibitors, antidepressants, PPIs or statins);

• A binary variable capturing whether a patient was female or male;

• The age of the patient at the time of generic/branded substitution (divided in four quantiles);

• A binary variable defining whether the prescription referred to the patient's first prescription from the specific drug class;

• A variable capturing the prescription history of a patient, defined as the number of all prescriptions from the specific drug class in the last six months;

• A variable capturing the patient's ratio of branded versus all prescriptions for the specific drug class in the last six months;

• A categorical variable capturing the pharmacy ownership status and distinguishing between:

○ an individually owned pharmacy;

○ a pharmacy belonging (among other pharmacies) to a multiple pharmacies' owner;

○ a pharmacy belonging to a partnership; and

○ a pharmacy belonging to a pharmacy chain;

• The duration after patent expiry (in months); i.e. the time between patent expiry and the specific prescription considered.

### Statistical Analysis

A Generalized Linear Mixed Model (GLMM) with crossed random effects [13-15] was applied in order to model the effect of the independent variables on the likelihood of generic and branded substitution by the pharmacist. The hierarchical structure of our dataset, with prescriptions originating from 4,417 different patients and 26 different pharmacies, necessitated the use of a multilevel, random effect approach in the modeling of the covariates' effects. Random effects accounted for the fact that prescriptions with the same observable independent factors, but filled by different pharmacies (yet, of the same ownership status) or referring to different patients (yet of the same age category and sex), may have different probabilities of being substituted. Furthermore, the non-nested structure of the random effects (since every patient can logically pick up drugs from various pharmacies) urged the use of an approach which would allow us to include patients and pharmacists as crossed independent random effects. Hence, for the *i^th ^*time that the *j^th ^*individual received a drug from the *k^th ^*drug class by the *l^th ^*pharmacy, we defined the binary responses:

and

By denoting this in terms of probabilities (Pr) - in particular, *π_ijkl _*= Pr(*y_ijkl _*= 1) and *ξ_ijkl _*= Pr(*h_ijkl _*= 1) - the models for generic and branded substitution can be formulated as(1)(2)

Where *X_ijkl _*and *Z_ijkl _*contain the independent covariates used in both specifications, *β *and *γ *are the fixed effect parameters and *α_j_, δ_j _*and *u_l_,, e_l _*the patient and pharmacist random effects parameters, respectively. In order for equations (1) and (2) to be identifiable we assumed that each random effects parameter follows a normal distribution with mean zero and variance *σ_α_, σ_δ_, σ_u _*and *σ_e _*respectively.

The assumption of normality for the two random effect parameters was tested with the help of *QQ *plots and the Shapiro-Wilk normality test [16]. Comparison among models with different covariate sets was based on likelihood ratio tests (LRT) [17]. The Somers' *D_xy _*criterion, a transformation of the level of concordance criterion, was calculated for every model to further assess the goodness of the model fit [18]. The closer the value of *D_xy _*was to one, the better the fit of the model was. Finally, the Cook's distance for GLMMs was estimated in order to check the influence of the individual components of the random effects (pharmacies or patients) on the parameter estimates [19]. In particular, components whose Cook's distance value exceeded that of 4/*n *(*n *being the number of individual components within every random effect) were considered to be influencing the model estimates more strongly than should be expected [20].

For the estimation of the GLMM we used the package "lme4" from the freely available statistical software R (version 2.10.0) [21,22].

## Results

### Subject Characteristics

**Figure 1:**
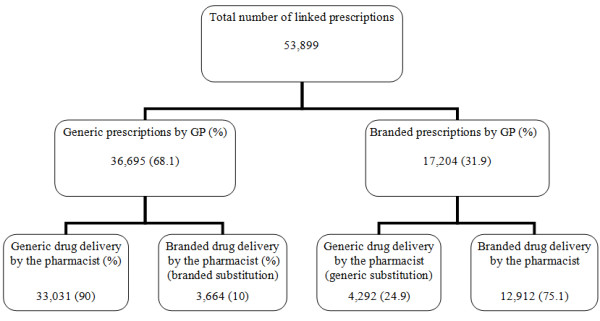
**Number of linked prescriptions (pharmacy and GP database) and percentages (between brackets) of branded - generic prescribing**.

The linked prescription data consisted of 53,899 prescriptions originating from 4,417 subjects. Of these prescriptions 36,695 (68.1%) were generically prescribed by the GP. From the rest 17,204 (31.9%) branded prescriptions, 4,292 were generically substituted by the pharmacist; i.e. 24.9% of branded was generically substituted. Also, out of all generic prescriptions 3,664 (10%) were filled with a branded drug by the pharmacist (Figure 1).

Table 2 presents the characteristics of the prescriptions used in the branded and generic substitution analysis, respectively.

**Table 2:** Summary of linked prescriptions (pharmacy and GP database) for the two analyses: branded prescriptions that can be potentially substituted generically (generic substitution analysis) and generic prescriptions that can be potentially substituted by a branded product (branded substitution analysis)

	Generic substitution analysis	Branded substitution analysis
No. of linked prescriptions	17,204	36,695
No. of prescriptions (%) on:		
• ACE inhibitors	6,068 (35.27)	10,259 (27.96)
• Antidepressants	4,626 (26.9)	11,760 (32.04)
• PPIs	3,788 (22.03)	8,187 (22.32)
• Statins	2,721 (15.81)	6,491 (17.68)
Average prescription- delivery lag in days (SD)	0.48 (0.84)	0.41 (0.91)
Average duration after patent expiry in months (SD)	27.47 (12.83)	35.07 (19.84)
No. of pharmacies	26	26
Individual pharmacy ^a^	18	19
Pharmacy in a partnership ^a^	8	9
Pharmacy in a chain ^a^	5	7
No. sample patients	1,423	3,855
Female patients (%)	819 (57.6)	2,215 (57.5)
Average age in years (SD)	61.05 (15.15)	57.96 (16.6)

^a ^Pharmacy ownership status can vary over time for each pharmacy

SD = standard deviation

### GLMM results for generic substitution

The estimates of the applied GLMMs on the probability of generic and branded substitution are presented in Table 3. The fixed effects estimates indicate that the probability of generic substitution is negatively associated with a high proportion of prior brand use (β's varying from -3.210 to -5.952, with *P *< 0.001 in both cases; see Table 3) and receiving a prescription further beyond the patent expiry date (*β *= -0.011, *P *= 0.002). If a prescription refers to a drug belonging to a drug class that is prescribed for the first time to the patient, the likelihood of generic substitution is smaller (*β *= -2.582, *P *< 0.001). Being dispensed a prescription from a pharmacy belonging to a partnership (*β *= 0.954, *P *= 0.046) or a chain (*β *= 0.687*, P *< 0.001), to a patient having more experience on the use of drugs from the specific drug class (*β *= 0.076, *P *< 0.001) and being older than the baseline age group are all positively associated with the probability of generic substitution. Finally, prescriptions referring to antidepressants or PPIs are less likely to be generically substituted (*β *= -0.579, *P *= 0.004 and *β *= -1.085, *P*< 0.001 respectively), compared to prescriptions of ACE inhibitors. The opposite holds true for statins (β = 0.574, *P *= 0.001), again as compared to ACE inhibitors.

**Table 3:** Estimates of the generalized linear mixed models on the probability of generic and branded substitution

	Generic substitution		Branded substitution	
	
	Log Odds (*β*)	*P*-value	Log Odds (*β*)	*P*-value
*Fixed effects estimates*				
Intercept	4.262	< 0.001	-6.978	< 0.001
Prescriptions to female patients	-0.163	0.284	0.095	0.391
First prescription in the drug class	-2.582	< 0.001	2.752	< 0.001
Prescription history	0.076	< 0.001	0.016	0.361
Branded ratio				
Branded ratio [0 - 0.33)	baseline			
Branded ratio [0.33 - 0.66)	-3.210	< 0.001	2.960	< 0.001
Branded ratio [0.66 - 1]	-5.952	< 0.001	5.809	< 0.001
Pharmacy property status				
Individually owned pharmacy	baseline			
Pharmacy in a partnership	0.954	0.046	-1.752	< 0.001
Pharmacy in a chain	0.687	< 0.001	-0.605	< 0.001
Duration after patent expiry (months)	-0.011	0.002	-0.046	< 0.001
Age				
Age [16-31]	baseline			
Age (31-46]	0.568	< 0.007	-0.399	0.038
Age (46-52]	0.546	0.012	-0.146	0.307
Age (52-88]	0.474	0.028	-0.153	0.262
Drug group				
ACE Inhibitors	baseline			
Antidepressants	-0.579	0.004	1.846	< 0.001
PPIs	-1.085	< 0.001	0.986	< 0.001
Statins	0.574	0.001	-0.634	< 0.001
*Random effects estimates*				
Pharmacy-level residual variance	1.915		0.350	
Patient- level residual variance	2.346		1.879	
Somers *D_xy _*criterion	0.985		0.981	

The LRTs comparing the full model of Table 3 against the reduced models, where each of the random effects are removed gradually (not shown here), established the significance of both random terms. Hence, there is evidence that the probability of generic substitution varies from patient to patient and from pharmacy to pharmacy. The assumption of normality of the random effect term was rejected for both pharmacy and patient random effect terms (*P *< 0.001 for both SW tests). QQ plots (not shown here) revealed that the distribution of the random effects was leptokurtic (i.e more peaked around the mean) but concentrated around zero. This implies that the misspecification might have negatively affected the efficiency of the fixed effect estimates but not their consistency [17,23].

Estimation of the Cook's distance on the full set of covariates showed that only one pharmacy marginally exceeded the cut-off point. No significant differences on the fixed effect estimates however were observed after re-estimation of the model on data that excluded any prescriptions from this specific pharmacy. Furthermore, after the re-estimations the new variance of the pharmacy-related random effect term was significantly smaller and there was no evidence to reject the assumption of normality through the SW test. Finally, the Somers *D_xy_*criterion gave evidence of a very good fit for the model (*D_xy _*= 0.985).

### GLMM results for branded substitution

As far as the branded substitution is concerned ( also shown in Table 3), results that generally mirrored those for generic substitution were found. In particular, a high proportion of prior brand use (*β's *ranging from 2.968 to 5.809, with *P *< 0.001 in both cases) as well as whether the patient is dispensed a prescription for the specific drug class for the first time (*β *= 2.752, *P *< 0.001) were found to be positively related with the probability of branded substitution. Oppositely, the distance from patent expiry (*β *= -0.046, *P *< 0.001) as well as whether the dispensing pharmacy belongs to a chain (*β *= -0.605, *P *< 0.001) or a partnership (*β *= -1.752, *P *< 0.001) were found to be negatively related to the probability of branded substitution. Generic antidepressant and PPI prescriptions were more likely to be substituted with a branded drug in comparison to ACE inhibitor prescriptions (β = 1.846, *P *< 0.001 and β = 0.986, *P *< 0.001, respectively). The opposite was found for statin prescriptions (β = -0.634, P < 0.001).

Again, the LRTs showed evidence of significant heterogeneity in the probability of branded substitution within patients and pharmacies. Cook's distances did not exceed the cut off point for any pharmacy or patient and the SW test found no evidence to reject the hypothesis of normality for the pharmacy random effect terms (SW test *P *= 0.627). However, the normality hypothesis for the patient random effects was rejected (SW test *P *< 0.001). Again, our results are likely to be conservative in terms of significance due to this violation, but not biased in terms of effect size [17,23].

Based on the Somers *D_xy _*criterion, the model fitted very adequately to the branded substitution data (*D_xy _*= 0.981).

## Discussion

In this study we analyzed the route of a prescription, from the moment of a treatment decision by the GP to the drug delivery by the pharmacist, with a specific interest in generic and branded substitution. In particular, we analyzed the behaviour of the pharmacist with regard to generic and branded substitution by using information related to patients' and pharmacists' characteristics and the specific timing of the prescriptions and deliveries. For this purpose, prescription-level information from both a GPs' and a pharmacies' database was merged.

We found that two of every three prescriptions were generically prescribed by the GP, while for 32% of the prescriptions the GPs prescribed the branded drug. The relatively high proportion of prescriptions written using the brand name could possibly indicate the effect of pharmaceutical detailing and marketing efforts and routine prescribing of branded drugs by the GPs [24]. We are currently directing one of the further researches into this aspect.

Following the prescription until delivery of the drug by the pharmacist, we noticed that generic substitution occurs in 8% of the total prescriptions (25% of the branded prescriptions), only marginally more often than branded substitution (6.7% of the total prescriptions, 10% of the generic prescriptions). We estimated generalized linear mixed models (GLMMs) for both types of substitution in an effort to identify the characteristics that may lead the pharmacist towards generic or branded substitution. Application of the GLMMs yielded results regarding duration from patent expiry that were in accordance with prior studies [7,25].

In particular, the probability of generic substitution for a prescription was found to be significantly and positively related to the experience of the patient on the use of drugs of the same drug class but negatively related to the proportion of prior branded use in this drug class. Obviously, it is less likely for a patient who has routinely been delivered the branded drug in the past, and is again prescribed a branded, to have his prescription generically substituted by the pharmacist. The opposite holds true for the case of branded substitution where prior branded use seems to positively affect the probability of branded substitution. In the case of the first prescription for any user, pharmacists seem to be more reluctant regarding generic substitution. The pharmacist, possibly following the patients' perception that branded drugs are generally safer and more effective, prefers to either deliver a branded prescription or even substitute a generic one with the branded.

The higher likelihood for both generic and branded substitution close to the time of patent expiry can possibly be explained by the dynamic process of generic diffusion. After the patent expiry date, GPs may still prescribe the branded drug but the stock of the pharmacist might already be replaced with generic alternatives, enhancing the impetus for generic substitution. Oppositely, the generic might be prescribed while there is still branded stock available and/or lack of information in the pharmacy regarding the existence, efficacy and safety of generic alternatives. In general, we notice that substitution by the pharmacist is very much concentrated in the period where the generic drug starts diffusing in the market.

The lower probability for branded substitution, in combination with the higher probability of generic substitution of prescriptions dispensed by pharmacies belonging to a chain or a partnership might be related to the fact that these organizations have a stricter policy as well as additional incentives towards generic use [26]. Also, bulk orders of generic drugs by chain pharmacies may result in higher discounts from the generic manufacturers and, hence, into lower generic acquisition costs [27].

Finally, differences were identified between substitution patterns of different drug groups. Antidepressants and PPIs were found to be less often generically substituted and more frequently substituted from generic alternative to branded, compared to ACE inhibitors and statins. The reduced probability for the class of antidepressants could possibly be attributed to concerns of patients on the effectiveness of the generic alternative. Furthermore, the patient expiry of the branded version of paroxetine was followed by specific patent extensions and legal battles from the producing pharmaceutical company in order to maintain the branded market share with a potentially substantial effect on substitution patterns [28]. A similar cause might be attributed to the lower probability of generic substitution on PPIs. The branded manufacturer of omeprazole introduced a new formulation of the drug that, in combination with extensive marketing efforts, maintained a large share of the omeprazole market [29]. Furthermore, concerns have been raised on the in vitro dissolution profile and kinetic properties of generic omeprazole compared to the branded drug [30].

Through the application of a GLMM approach, we detected a significant amount of heterogeneity in the behaviour of pharmacists towards, mainly generic, substitution. Except from the analysed characteristic of the pharmacy status, which was available to us, specific location and competition factors may be expected to additionally influence the decision of the pharmacist on substitution. A pharmacy in a more remote district which might enjoy a certain monopoly on drug distribution over the area will possibly be more inclined to substitution if this is financial-economically attractive. Oppositely, pharmacy competition within a single city might oblige pharmacists to be more elastic towards specific patient requests, for example, regarding preferences for staying on the branded product with which potentially a lot of experience already exists. Also, no information on potential discounts achieved by pharmacies and other financial benefits that might co-determine pharmacists' behaviour were available to us. There was also considerable evidence found regarding the unobserved variation in the substitution probability among different patients. This was expected since patients behave differently when confronted with the decision of substitution, depending on characteristics that are impossible to be identified through our dataset such as tolerability of the generic alternative, existence of comorbidities and attitudes towards drug use. Such heterogeneity in behaviour of pharmacists and patients, potentially related to above mentioned factors unobserved in our database, provides a strong rationale to use the random effects model as we did here.

To our knowledge, this is the first paper that describes the extent and nature of generic and branded substitution by pharmacists in the Netherlands. Our efforts to explicitly identify factors that might affect the likelihood of these substitutions may help in designing specific strategies to achieve general goals in this area, often concerning the increase of generic and discouragement of branded substitution. In respect, our findings indicate that policies enhancing generic substitution could be targeted to increase generic use at first prescription, for those patients with a long history on the branded drug, the relatively young patients and in individually-owned pharmacies. As these aspects are mirrored in our analysis on branded substitution, the same types of prescriptions, patient groups and pharmacies should be targeted for discouraging branded substitution. Explicit information on safety and efficacy of the generic PPIs and antidepressants could help raising levels of generic substitution (decreasing levels of branded substitution) to levels achieved for the other drug groups investigated namely of statins and ACE-inhibitors.

One caveat in our analysis is the lack of information concerning the capability of the pharmacist to dispense the generic drug instead of the branded, as some of the prescriptions explicitly indicate that generic substitution should not be performed. Another important factor of influence on which our databases offer no information is the monetary incentives that pharmacists are offered, most often in the form of discounts, from the pharmaceutical industry. Given that generic manufacturers have been evidently found to offer greater discounts than branded manufacturers [5,31] it could however be hypothesized that the presence of discounts results into higher generic substitution levels, yet the data to actually analyse this currently lack us.

Furthermore, despite the use of a mixed model to correct for unobserved heterogeneity, we might still lack important information on other potential explanatory variables such as pharmacy stock sizes, regional insurance companies' rules and incentives for substitution (e.g. preference policy) and the intensity of detailing. Further research is directed towards providing more insight regarding these yet "unobserved" variables.

Another interesting extension of the current research would be an analysis on the effect of patent expiry on therapeutic substitution. Although Klok et.al [30] have already identified a positive effect of the patent expiry of branded omeprazole on therapeutic substitution within the PPI group, investigation in a larger number of drug classes would give a more general impression of the effect of patent expiries on therapeutic substitution.

## Conclusions

Overall our findings suggest that even though generic delivery is strongly connected to generic prescribing, the event of generic substitution, when it occurs, is mainly affected by the type of the pharmacy ownership, the timing of the prescription, the type of the drug and the patient's age and experience with the drug. The development of policies to further contain Dutch drug costs, using generic substitution as an instrument for this, could benefit from this knowledge, for example, by specifically targeting types of pharmacies, patients or drug classes.

## Authors' contributions

PP the designed the study in detail, performed the statistical analysis and drafted the manuscript. WJV participated in the design of the study, in the linking procedure and read and improved the manuscript. JHB participated in the linking procedure and read and improved the manuscript. MJP conceived the study, participated in its design and coordination and helped to draft the manuscript. All authors read and approved the final manuscript.

## Competing interests

The authors declare that they have no competing interests.

## Pre-publication history

The pre-publication history for this paper can be accessed here:

http://www.biomedcentral.com/1472-6963/11/89/prepub
